# Effects of Focal Ionizing Radiation of the Squid Stellate Ganglion on Synaptic and Axonal Transmission in the Giant-Fiber Pathway

**DOI:** 10.7759/cureus.13110

**Published:** 2021-02-03

**Authors:** William F Gilly, P. Teal, Edward E Graves, Jackei Lo, M. Bret Schneider, Reese Zasio, John R Adler

**Affiliations:** 1 Biology, Hopkins Marine Station, Stanford University, Pacific Grove, USA; 2 Radiation Oncology, Stanford University School of Medicine, Stanford, USA; 3 Radiation Oncology, Stanford Health Care, Stanford, USA; 4 Neurosurgery, Stanford University School of Medicine, Stanford, USA; 5 Psychiatry, Stanford University School of Medicine, Stanford, USA; 6 Veterinary Service Center, Stanford University School of Medicine, Stanford, USA; 7 Radiation Oncology, Stanford University Medical Center, Stanford, USA

**Keywords:** irradiation, squid, axon, synapse, action potential, potassium conductance, glutamatergic synaptic transmission

## Abstract

Ionizing radiation is clinically used to treat neurological problems and reduce pathological levels of neural activity in the brain, but its cellular-level mechanisms are not well understood. Although spontaneous and stimulated synaptic activity has been produced in rodents by clinically and environmentally relevant doses of radiation, the effects on basic excitability properties of neurons have seldom been reported. This study examined the effects of focused ionizing radiation on synaptic transmission and action potential generation in the squid giant-fiber system, which includes the giant synapse between a secondary interneuron and the tertiary giant motor axons. Radiation of 140-300 Gy was delivered to a stellate ganglion of a living squid over several minutes, with the contralateral stellate ganglion serving as an internal control. No qualitative changes in the efficacy of synaptic transmission were noted in conjunction with stimulation of the input to the giant synapse, although in one irradiated ganglion, the refractory period increased from about 5 ms to more than 45 seconds. Small but significant changes in the action potential recorded from the giant motor axon in response to electrical stimulation were associated with an increased maximum rate of fall and a shortened action potential duration. Other action-potential parameters, including resting potential, overshoot, the maximum rate of the rise, and the refractory period were not significantly changed. Attempts to account for the observed changes in the action potential were carried through a Hodgkin-Huxley model of the action potential. This approach suggests that an increase in the maximum voltage-gated potassium conductance of about 50% mimics the action potential shortening and increased rate of fall that was experimentally observed. We propose that such an effect could result from phosphorylation of squid potassium channels.

## Introduction

Sub-ablative doses of focused ionizing radiation delivered to brain circuit nodes have been proposed as a basis of therapy for some intractable conditions. While there is evidence supporting a neuromodulation effect of radiation, the mechanisms of action are not well understood. Remediation of neuropathology using irradiation is often attributed to DNA damage, but it is not clear how non-ablative procedures would mediate observed effects on non-dividing neurons in this manner. Proposed alternative mechanisms include vascular effects including edema and hypoxia, glial effects, and direct neuronal effects, including alteration of synapses and axonal membranes [1].

Mechanistic studies of neuronal sensitivity to irradiation at the cellular level have been limited. For example, focused irradiation of 20 and 40 Gray (Gy) delivered to the hippocampal region in rats using a gamma knife substantially decreased penicillin-induced epileptiform spiking in hippocampal neurons several weeks after exposure, but not before [2]. Doses in this range delivered via a proton beam did not appear to alter synaptic input and excitability of granule cells in hippocampal slices, but stronger doses (90-130 CGE) reduced activity and caused necrosis [3]. Older studies using similar doses of radiation to isolated hippocampal slices from guinea pigs reported a variety of effects related to the synaptic activity, which were observed immediately after irradiation [4,5].

Effects of low-level whole-body radiation (0.05-1.0 Gy) from proton [6] and neutron [7] sources have been investigated in mice at the organismal and cellular level with effects generally being assayed 6-12 months after exposure. Both cited studies found an increase in resting potential (i.e., more negative) in individual neurons and a general decrease in spiking and excitatory synaptic activity in hippocampal slices consistent with a general decrease in activity. However, the properties of action potentials in individual neurons were not affected. Similar results were found with chronic low-level neutron-irradiation after 0.001 Gy/day was delivered for six months with assays being carried out six months later [8].

Although ionizing radiation may suppress neuronal activity at the cellular level, the relevant biophysical or molecular mechanisms have not been identified, even in the case of acute exposure that might generate rapidly detectable effects. Exploring these mechanisms is complicated by the fact any neuron is surrounded by an in vivo environment that includes other neurons of a variety of types and synaptic interconnections, glia, and blood vessels. And excitability properties of individual neurons will vary, even within a given subtype. Furthermore, the neuron itself consists of anatomically and functionally discrete regions, including the cell body, dendrites, axon, and synaptic terminals.

No experimental model system allows isolating all these components, but classical preparations based on “identified” giant neurons of invertebrates, including the squid giant-fiber pathway, offer some unique advantages for cellular-level analyses of synaptic and axonal function. Squid “giant axons” as studied by Hodgkin and Huxley [9] are motor axons in loliginid squid that constitute the terminal, third-order (3º) neuronal component in the giant-fiber pathway that controls jet-propelled swimming, an important feature of the escape response [10]. These non-myelinated axons, about 10 on each side of the squid, arise in the giant-fiber lobe of the ipsilateral stellate ganglion and project into the mantle musculature. Each giant axon is contacted within the stellate ganglion by a second-order (2º) giant axon that projects from a giant interneuron in the brain via the pallial nerve. These excitatory “giant synapses” are visible in the living ganglion, and transmission at the synapse with the hindmost (and largest) giant axon has been extensively studied [11,12]. The extremely large size of pre- and post-synaptic elements and relatively simple anatomy greatly facilitate experimental approaches using intracellular recording methods, and for these reasons, classical, seminal work on axonal and synaptic function utilized this preparation.

In this study, we sought to take advantage of the squid giant axon-synapse preparation to ascertain the acute effects of focused ionizing radiation on synaptic and axonal transmission. Living squid were subjected to focal irradiation (140-300 Gy over 139-298 seconds) delivered to the stellate ganglion on one side, with the contralateral ganglion serving as a control. Electrophysiological experiments were used to assay synaptic and axonal function within 24 hours. We found that irradiation leads to a small, but significant, shortening of the action potential and increased rate of repolarization in the 3º giant axon. Attempts to model this effect using a Hodgkin-Huxley approach suggest that modulation of the voltage-dependent potassium (K) channel system may be a relevant mechanism.

## Materials and methods

Animals and irradiation

Squid were collected during April-May 2019 by jigging in Monterey Bay and placed immediately into a large cooler with aerated seawater. The squid were then transported about one mile to Hopkins Marine Station where they were maintained in a circular tank (1 m x 2.5 m) with flow-through seawater at ambient temperature (14-15 ºC) provided by the Monterey Bay Aquarium. Squid were fed goldfish or rosy red minnows daily and were held for no longer than nine days before use.

Healthy squid with no obvious physical damage were selected and anesthetized in 1-2% ethanol in seawater at ambient temperature, and a small piece of skin was removed over the left stellate ganglion with fine Vannas-type scissors using a 10x magnifying visor. A 20 g sterile needle was dipped in black ink and used to mark a spot on the mantle muscle (2-3 mm thick) directly over the ganglion to assist in positioning the radiation beam. The squid were then revived in fresh seawater, placed in a cooler with chilled, aerated seawater, and transported by vehicle to the Stanford School of Medicine where they were irradiated in the Veterinary Service Center (VSC) facility using a 225 KV radiation tube and associated gantry (Kimtron Inc., Oxford, CT). Calibration was carried out using Gafchromic EBT3 film (Ashland Advanced Materials, Bridgewater, NJ), and the dose was determined to be 60.37 Gy per minute at the selected treatment distance.

**Figure 1:**
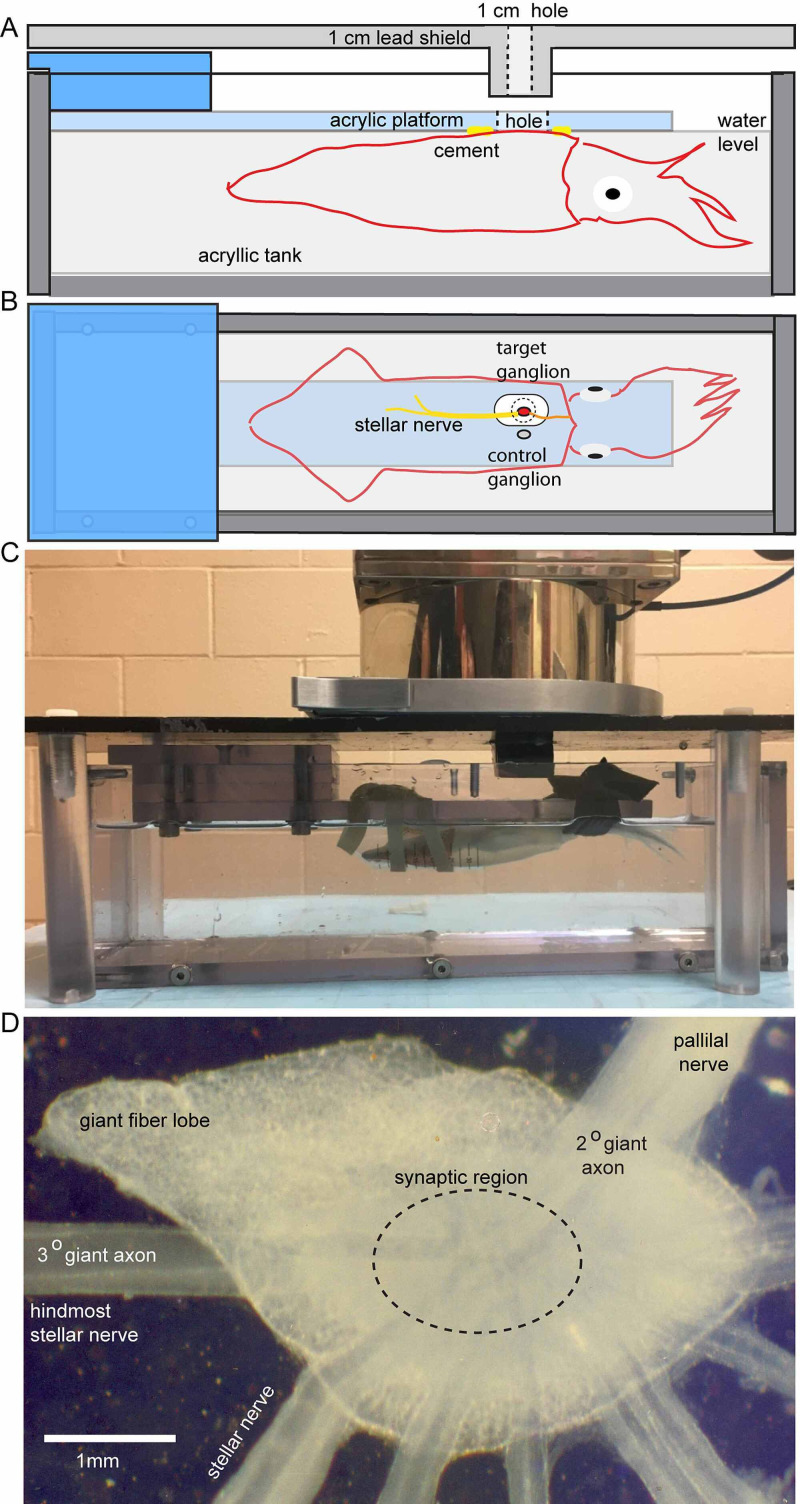
Chamber for holding the living squid during irradiation of a stellate ganglion A. Side view of the watertight acrylic tank with squid attached to an acrylic support-platform with cyanoacrylate cement. A 1-cm thick lead shield over the top of the tank had a 9-mm diameter hole that was positioned over the target stellate ganglion for irradiation. An additional 2-cm thick ring of lead (9 mm internal diameter) was aligned with the hole in the cover to confine the beam to the target area. Squid of <15 cm mantle length could be accommodated in this device B. Top view of the suspended squid, with the lead cover removed, showing the hole in the support platform positioned over the target stellate ganglion. The contralateral ganglion was not exposed to radiation and served as an internal control C. Photograph of a living squid prepared for irradiation. In this case, a 50-ml Falcon tube was slipped over the mantle, and spandex ties were cinched around the tube and the squid’s head to the support platform to minimize movement during irradiation D. Excised living ganglion showing the hindmost 3° giant motor axon in a stellar nerve and the pallial nerve that contains the 2° giant axon. The giant synapse forms between these elements in the synaptic region within the neuropil of the ganglion (dashed ellipse). The view is from the ventral surface with posterior to the left and medial on top

Prior to irradiation, the squid was removed from the cooler and restrained in a seawater-filled chamber by attaching the dorsal surface of the mantle to an acrylic platform using cyanoacrylate cement [13], with the left stellate ganglion positioned directly beneath a 9 mm circular aperture in the lead cover plate (3 cm thick in the area over the ganglion) of the chamber that served to shield areas other than the ganglion form radiation (Figure 1A, Figure 1B). The chamber was then placed beneath the X-ray source, and its position was adjusted manually to align the left stellate ganglion of the suspended squid with the X-ray source (Figure 1C) at a distance D of 21.977 cm to the target. Radiation was then delivered to the stellate ganglion using a single KV X-ray beam at 140-300 Gy.

Following irradiation, the squid was carefully removed from the acrylic restraining platform, returned to the cooler of chilled, aerated seawater, and returned by vehicle to Hopkins Marine Station where it was maintained in isolation in a smaller circular tank (1 m x 1 m) with flow-through seawater. Squid were used for physiological experiments on the next day.

A total of eight squid were studied in this manner and successfully irradiated (Table 1). Mortality associated with transport and irradiation was 20% (3/15 squid).

**Table 1:** Summary of experiments with irradiated squid Entries with an asterisk (*) indicate individual (numbered) squid in which one stellate ganglion was irradiated in the living animal, and the contralateral ganglion served as a control (0 Gy dose). Useful data collected with stimulation of the presynaptic 2º giant axon in the pallial nerve (2º stim.) or with stimulation of the postsynaptic 3º giant axon in the stellar nerve (3º stim.) are indicated by a plus sign (+). A low resting potential in the 3º axon is indicated by a minus sign (-); these data were excluded from further analysis. Preparations that were visually determined to be damaged during dissection are indicated by x A total of four 3º-stimulation experiments were successful with irradiated and control ganglia in the same squid (Squid 2, 5, 6, 7). Four preparations yielded useful 2º-stimulation data with irradiated ganglia, but control data from the same squid were only available in one case

Squid	Ganglion	Dose (Gy)	2º stim.	3º stim.
8	31MAY19C	200	x	x
8	31MAY19D	0	x	+
7	31MAY9A*	300	+	+
7	31MAY9B*	0	+	+
6	14MAY19C*	200	+	+
6	14MAY19D*	0	x	+
5	14MAY19A*	200	+	+
5	14MAY19B*	0	x	+
4	08MAY19A	200	-	-
4	08MAY19B	0	+	+
3	04MAY19A	200	x	x
3	04MAY19B	0	+	-
2	03MAY19A*	140	+	+
2	03MAY19B*	0	x	+
1	01MAY19A	140	x	x
1	01MAY19B	0	-	-
0	26APR18B	0	+	-
	Total n	X-ray	4	4
		Control	4	6
		In same squid	1	4

Squid are not considered to be vertebrate animals by the National Institutes of Health (NIH) or Stanford University definitions, but we nonetheless supplied details regarding husbandry and experimental protocols for these animals used in this study to the Stanford University APLAC (IACUC) committee. Feeding of live fish to squid is permitted under Stanford University APLAC Protocol 10643, “Fish as food in husbandry of molluscs,” approved for the period of 09/21/2017 to 09/18/2020.

Electrophysiology 

Squid used for electrophysiology were euthanized by rapid decapitation, taking care not to damage the posterior region of the head. This region of the head and about 8 cm of the mantle (opened along the ventral midline) was pinned out ventral side up in a Sylgard-filled dish in chilled, aerated seawater drawn from a reservoir kept at 4º C with bubbling air in a refrigerator. This seawater was replaced regularly during the following procedures.

Left and right pallial nerves were first exposed under stereomicroscopic examination and ligated with 6-0 surgical silk near their emergence from the skull. Left and right hindmost stellar nerves were then ligated about 3 cm distal to the stellate ganglion. Ganglia with attached pallial and stellar nerves were then carefully exposed on each side, removed, and placed into a small beaker of chilled, aerated seawater. Each ganglion and associated nerves were then pinned out for recording in a separate plastic petri dish (5.5 cm diameter) and placed in a refrigerator until use. In all cases, only the left stellate ganglion was irradiated, and the right ganglion served as a control. 

A ganglion for experimentation, similar to the preparation in Figure 1D, was mounted on the fixed-stage of an Olympus BH2 upright microscope equipped with a cooling stage to maintain the preparation at 15 ºC and viewed through a long-working distance objective (Nikon Plan 2X). All recordings were carried out in filtered seawater.

Conventional intracellular electrodes were filled at 3 M KCl (resistance of ~10 Megohms) and connected via a silver wire coated with AgCl to an electrometer preamplifier (OC-725C: Warner Instruments, Hamden, CT). A pair of Ag:AgCl wires connected to the bath-clamp circuit of the preamplifier held the bath voltage at the ground. A glass extracellular focal-recording electrode with a tip diameter of ~160 µm was filled with seawater, and a differential voltage was recorded between an Ag:AgCl wire inside the pipette and another wound around the outside near the tip using a P55 AC-coupled amplifier (Grass Instruments, Astro-Med, Inc., Warwick, RI). A coaxial bipolar stimulating electrode (tungsten) with a tip diameter of ~200 µm (Rhodes Medical Instruments, Woodland Hills, CA) was used to stimulate either the pallial nerve upstream of the ganglion or the hindmost stellar nerve about 0.5 cm downstream of the ganglion (not illustrated) using an SD9 bipolar stimulator (Grass Instruments) triggered manually to generate a square wave of 30-35 µs duration. A synch-pulse generated by the stimulator triggered recording by the PC-based data-acquisition interface (Digidata 1320A) and pCLAMP 9 software (Molecular Devices, L.L.C., San Jose, CA). The sampling of all signals was at 50-100 KHz. Intracellular-voltage recordings were not filtered, and extracellular-voltage signals were low-pass filtered at 10 KHz and high-pass at 10 Hz using the preamplifier (gain of 100) before sampling. The analog 60 Hz line-filter was also employed.

The refractory period associated with action potential generation (recorded in the 3⁰ axon) was tested with stimulation of either the stellar nerve or the pallial nerve. In the latter case, the estimated refractory period would be expected to be that of the 2⁰ presynaptic axon with orthodromic propagation exciting the 3⁰ axon through the giant synapse. In these experiments, the interval between the two stimuli (measured between the midpoints of the two pulses) started at ~30 ms, and the threshold voltage (as displayed on the stimulator dial) was determined. The inter-pulse interval was then reduced, and the threshold was redetermined. This process was repeated until all-or-none action potential generation was no longer possible for the second stimulus.

Data analysis

Records obtained from 3º giant axons with resting potentials of <-60 mV were excluded from the analysis because the amplitude of the action potential decreased rapidly for resting potentials <~-55 mV. Data were analyzed using Clampfit software in the pCLAMP9 suite for the following parameters: resting potential (RP), the positive peak of action potential (Vpos), negative after-peak of action potential (Vneg), and duration at 50% amplitude (i.e., RP to Vpos) (half-width). These traces were also exported as text files into Igor Pro 6.3.7.2 (WaveMetrics, Lake Oswego, OR) for further analysis. The first time-derivative (dV/dt) was computed using Igor Pro to measure the maximum rate of the rise (dV/dt Rise) and fall (dV/dt Fall) [Figure 3D; and the second derivative (d^2^V/dt^2^)] was computed to estimate the time between the point of dV/dt Rise and dV/dt Fall. The time for the action potential to fall from 90% to 10% of its maximum amplitude (Vpos - resting potential) was also measured (90-10% Δt, not illustrated).

Statistical analysis of significance between irradiated and control groups of squid was carried out using an online statistics package (https://www.socscistatistics.com/) through a two-tailed T-test (https://www.socscistatistics.com/tests/studentttest/default2.aspx). Linear regression of dose-response data was carried out in Igor Pro, and the Pearson r coefficient generated by the fit was converted to p-values using the online calculator (https://www.socscistatistics.com/pvalues/pearsondistribution.aspx).

The refractory period was determined from a plot of inter-pulse interval values plotted on the ordinate with the corresponding voltages on the abscissa. Such plots were well fit by a single decaying exponential with a predicted horizontal asymptote that we define as the refractory period.

Action potential simulations

Simulated action potentials were calculated based on the Hodgkin-Huxley formulations using an online platform (HHsim 3.6: Graphical Hodgkin-Huxley Simulator) [14]. The default temperature for this simulation is 6.3 ⁰C. In order to best reproduce the properties of a control action potential as recorded in our experiments at 15 ⁰C (peak amplitude and maximum rates of the rise and fall), we found it necessary to adjust the following default parameters: temperature to 14 ⁰C, the concentration of external sodium from 440 mM to 470 mM, “fast sodium” maximum conductance from 120 µS to 92 µS; “delayed rectifier” maximum conductance from 36 µS to 32 µS, and “threshold for alpha n” from -55 mV to -53 mV. Computed data were downloaded from this program as text files and imported into Igor Pro, where all parameters discussed in the preceding paragraph were estimated. Because of the limited time-step in the model output (100 µs), calculated voltage traces were smoothed using two passes of the binomial filter in Igor Pro, dV/dt traces were filtered an additional 6X, and d^2^V/dt^2^ trace was filtered another 3x. This yielded adequate smoothing without compromising estimates of the relevant parameters.

## Results

Synaptic transmission at the giant synapse

**Figure 2:**
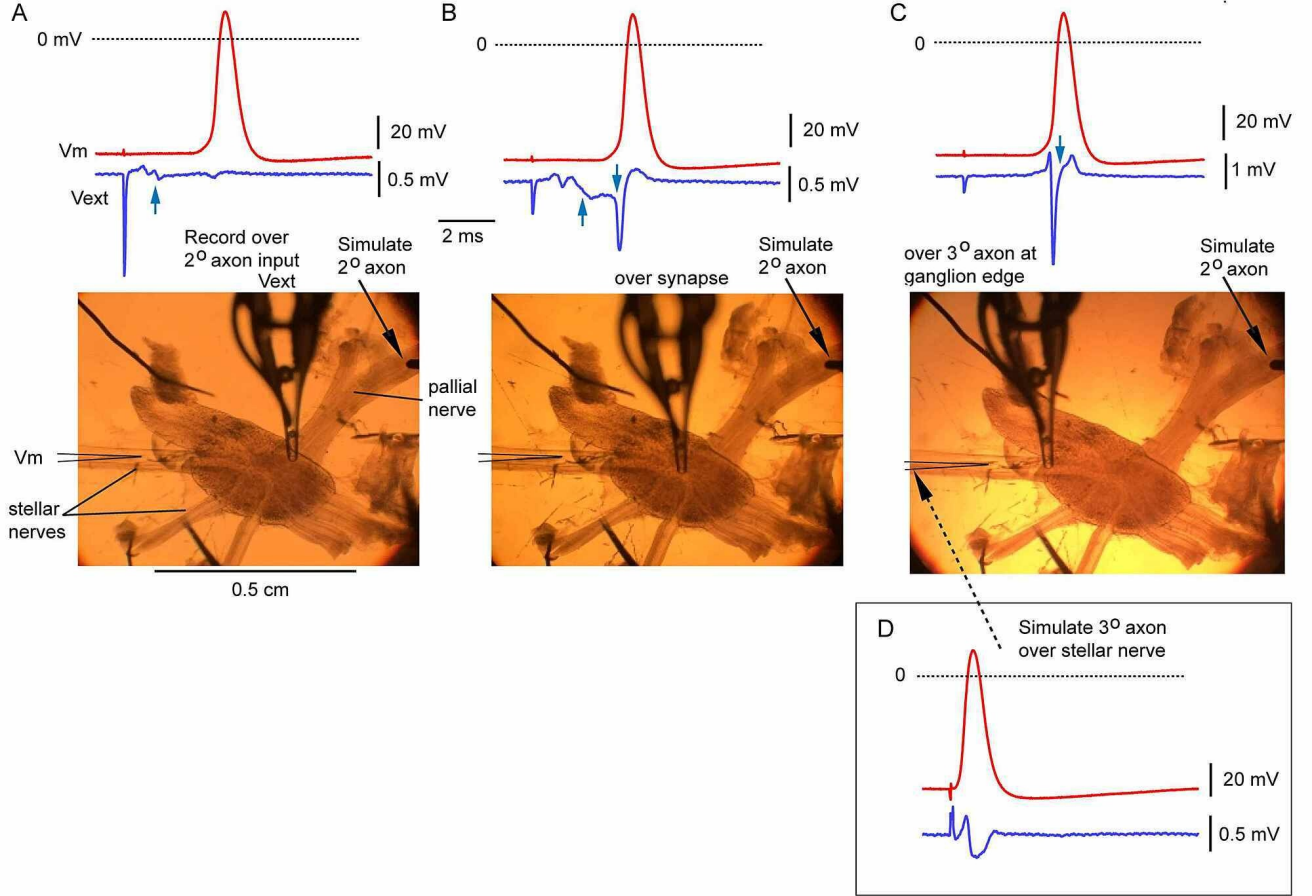
Extracellular recording to confirm the integrity of synaptic transmission and axonal excitability Membrane potential (Vm) was recorded with an intracellular microelectrode from the hindmost 3° giant axon ~1 mm downstream from the stellate ganglion. A glass focal-electrode was used to detect signals reflecting synaptic and axonal activity depending on where it was positioned. Stimulation of the pallial nerve stimulation results in an orthodromic spike in the 3° giant axon (A-C); stimulation of the stellar nerve downstream from the Vm recording site generates an antidromic spike (D) A. Focal electrode is positioned over the 2° axon where it enters the stellate ganglion. The small spike at short latency is due to activity in this axon (upward arrow) B. Focal electrode is positioned directly over the synaptic region in the stellate ganglion. The short-latency spike in the 2° axon pre-synaptic terminal is followed by a slower, monophasic negative way that corresponds to the post-synaptic inward current in the 3° axon (upward arrow). This wave is followed by a fast biphasic spike due to action-potential generation in the post-synaptic 3° element (downward arrow) C. Focal electrode is positioned over the 3° axon near the site of its emergence from the ganglion. The biphasic spike due to the propagating action potential is very large (downward arrow) D. Focal electrode is positioned in a manner like that in panel C, except stimulation, in this case, was via the stellar nerve downstream from the site of Vm recording. Propagation is therefore into the ganglion from the periphery (antidromic) Panels A-C were recorded from ganglion 04MAY19A from an irradiated squid. Panel D was recorded from ganglion 04MAY19B from the contralateral control ganglion of the same squid

Action-potential propagation in the 2º giant axon and transmission at the giant synapse in response to stimulation of the pallial nerve was confirmed by placing the extracellular focal-electrode over the entrance of the pallial nerve to the ganglion to detect the incoming spike in the presynaptic 2º giant axon (Figure 2A, arrow) or over the synaptic region of the ganglion to detect both this spike as well as a larger and slower, negative-going signal reflecting the excitatory junctional current at the giant synapses (Figure 2B, upward arrow) and a fast, biphasic spike due to the action potential in the postsynaptic 3º giant axons (downward arrow). Moving the focal electrode along the hindmost 3º giant axon downstream of the synaptic region reveals only the spike in that axon (Figure 2C).

Records in Figure 2 were obtained from an irradiated ganglion, and we saw no obvious sign of impaired synaptic transmission associated with irradiation in any of the ganglia successfully studied. In every successful preparation (four control and four irradiated ganglia, Table 1), a propagated action potential was recorded with the intracellular electrode in the 3º giant axon following stimulation of the pallial nerve, indicating successful synaptic transmission at the giant synapse. This was true for both control and irradiated ganglia.

The refractory period associated with excitation of the 2º axon and synaptic transmission, as assayed by action potentials recorded in the 3º giant axon had a value of 5.3 ±2.8 ms (n=3) in control ganglia versus 3.9 ms (n=2) in irradiated ganglia, and this difference is not significant (Table 2). Similar values were obtained in association with direct stimulation of the 3º giant axon (Table 2).

**Table 2:** Effects of irradiation on absolute refractory period estimated based on stimulation of the pallial nerve (2° stim.) or stellar nerve (3° stim.) No significant differences were found for irradiated and control preparations, but values from the anomalous preparation of 14MAY19A^3^* were not included in the mean value for irradiated samples. Values for 03MAY19A^2^ are overestimated because too few intervals were tested; they are not included in the calculation of the mean. The squid identification number is given in superscript SD: standard deviation

Experiment	Dose (Gy)	Refractory period 2º stim. (ms)	Refractory period 3º stim. (ms)
31MAY19B^7^	0	4.20	2.06
31MAY19D^8^	0		1.77
08MAY19B^4^	0	8.40	
04MAY19B^3^	0	3.20	3.49
03MAY19B^2^	0		3.73
Mean ±SD (n)		5.27 ±2.76 (3)	2.76 ±0.99 (4)
31MAY19A^7^	300	2.69	2.47
14MAY19A^3^*	200	45,000-60,000	
14MAY19C^6^	200		1.67
08MAY19A^4^	200	5.04	
03MAY19A^2^	140	<5.76	<7.50
Mean (n)		3.87 (2)	2.07 (2)
P-value		0.5751	0.4260

There was one significant exception to this generalization. In one irradiated ganglion (14MAY19A), a second action potential was not possible with presynaptic stimulation until 45-60 sec after the first stimulus (which led to a normal action potential in the 3º axon). During this period, a subthreshold EPSP was evident in the recording from the 3º giant axon, growing from ~7 mV peak amplitude for an inter-pulse interval of 20 ms to 12.5 mV at 25 s. The slow phase of this recovery was well fit by an exponential with a time constant of 19.4 s and a predicted amplitude of 14 mV at 60 s (not illustrated). This level is close to that at which a successful action potential showed an inflection at threshold during the EPSP, estimated to be ~15-16 mV positive to the resting potential. This anomalous preparation would thus appear to have a significant impairment in some aspect of excitatory transmission at the giant synapse, but this was not evident with a single stimulus.

Action potentials in the 3º giant axon

**Figure 3:**
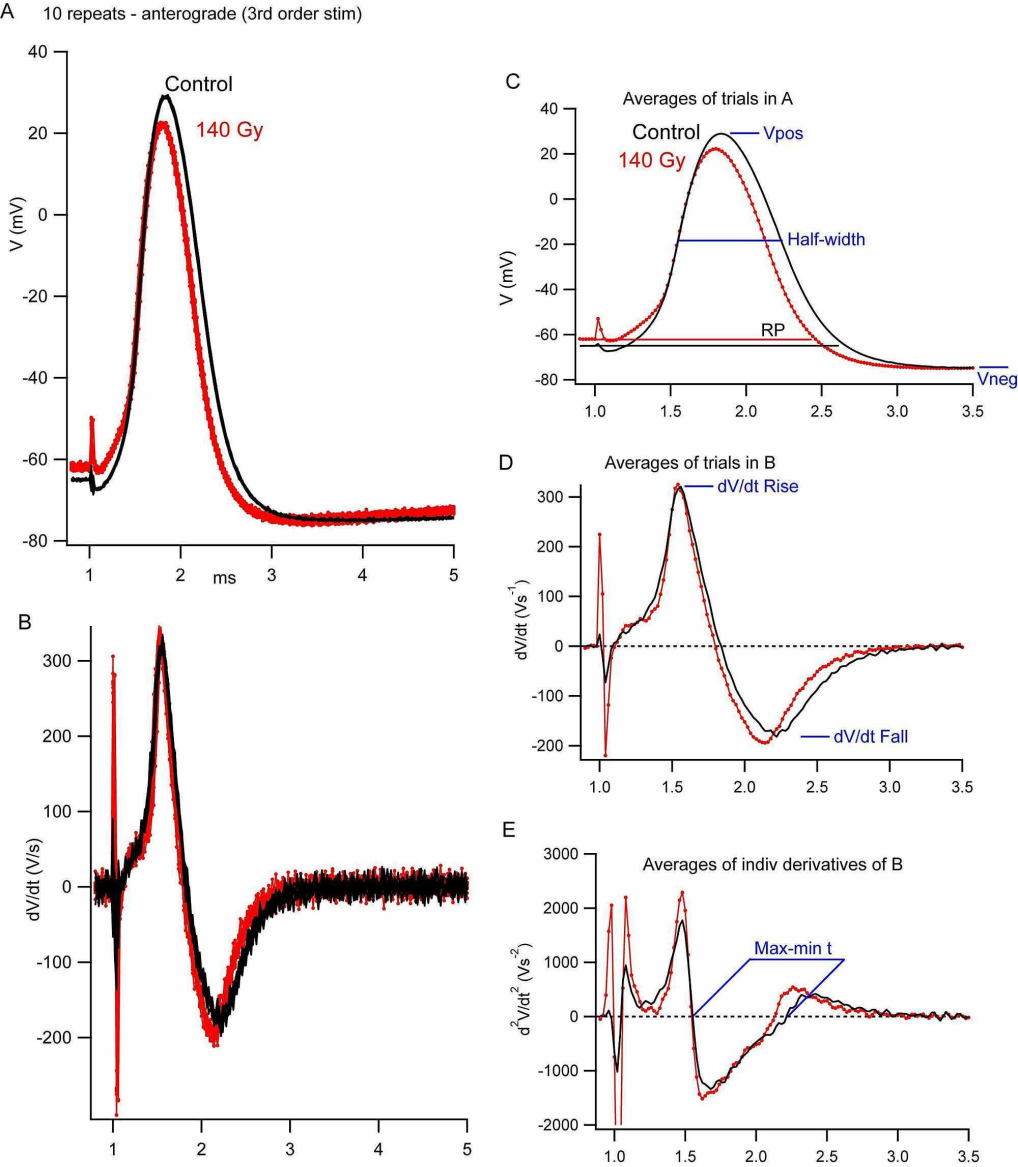
Effects of irradiation (140 Gy) on the action potential recorded in the 3º axon in response to stimulation of the stellar nerve (03MAY19A) A. 10 repeats obtained at a rate of about 1 Hz from an irradiated ganglion (red) and the contralateral control ganglion (black) from the same squid B. Time-derivatives of the action potentials in panel A to indicate the maximum value of dV/dt associated with the rising and falling phases of the action potential C. Averages of the Vm traces in panel A. Averaging is useful in this case because there is little variation in latency of individual spikes. Parameters used to characterize the action potential are indicated – overshoot (Vpos), duration at 50% amplitude (half-width), and undershoot (Vneg) D. Averages of the dV/dt traces in panel B. These records were used to measure the maximum rates of the rise (dV/dt Rise) and fall (dV/dt Fall) of the action potential E. Averages of individual second time-derivatives of the records in panel B. These records were used to measure the time between maximum rate of the rise and fall (min-max time) based on the times when the second-derivative trace crossed the zero baseline

Action potentials were recorded in the 3º giant axon in response to both indirect stimulations through the 2º giant axon in the pallial nerve via the giant synapse and direct stimulation of the 3º axon as described in Methods. Generally, 15-30 suprathreshold stimuli were delivered at an interval of ~1 s, and all responses were recorded (Figure 3A). All records were differentiated during analysis (Figure 3B) and analyzed separately in conjunction with the parameters discussed above and diagrammed in Figure 3C, Figure 3D, and Figure 3E. Each repetition thus generated a mean and standard deviation for each parameter with both 2º and 3º stimulation of each ganglion, although not all experiments were successful (Table 1). 

Records for 10 action potentials recorded in a control (black) and irradiated (red) ganglion from the same squid, in response to direct stimulation of the 3º axon, provide a qualitative picture of the general effect of irradiation that we observed - a slight shortening in the duration of the action potential (Figure 3A) and an increased maximum rate of fall (Figure 3B). As discussed below, the decrease in action potential amplitude (Vpos) was not reliably seen. These effects are more visually apparent if the averages for the two sets of records are examined in regard to half-width (Figure 3C), the maximum rate of fall (dV/dt Fall; Figure 3D), and the time between the maximum rate of the rise and fall (max-min time; Figure 3E).

A more quantitative analysis is summarized in Table 3 for 3º stimulation and in Table 4 for 2º stimulation. We consider the former data more reliable because of a larger number of successful experiments and the ability to compare data from control and irradiated ganglia in the same squid (n=4 versus vs. n=1 with 2º stimulation). Except for action potential undershoot (Vneg), all parameters associated with the falling phase of the action potential were significantly different in the pooled irradiation data (p<0.05). The maximum rate of fall is increased by ~15%, and a reduction of 11-13% is evident for the other parameters [half-width, max-min time, and the time to fall from 90% to 10% peak amplitude (90-10% rt)]. Resting potential, overshoot (Vpos), or maximum rate of the rise (dV/dt Rise) were not significantly different in control and irradiated ganglia.

**Table 3:** Effects of irradiation on the action potential in 3º giant axons with stellar-nerve simulation (retrograde propagation) Ganglion codes with superscript numerals (1-4) indicate experiments in which both the irradiated and (contralateral) non-irradiated stellate ganglia were tested in the same squid (paired)*. Two additional control experiments were conducted on other squid, and the averages of all six controls are also indicated (all)**. All values are mean ±SD; n indicates the number of action potentials analyzed. Only parameters associated with the downstroke of the action potential (^†^) were significantly different (p<0.05) in irradiated axons vs. control (paired or all) based on a two-tailed T-test

Ganglion	Dose (Gy)	n	RP (mV)	Vpos (mV)	Vneg (mV)	dV/dt Rise (V/s)	dV/dt Fall (V/s)	50% Δt (ms)	90-10% Δt (ms)	Max-min Δt (ms)
31MAY19B^1^	0	18	-62.6 ±0.5	28.8 ±0.4	-75.3 ±0.4	354.5 ±2.5	-214.8 ±1.6	0.636 ±0.001	0.459 ±0.002	0.662 ±0.010
14MAY19B^2^	0	19	-68.6 ±0.1	30.4 ±0.5	-81.3 ±0.2	379.6 ±6.9	-205.0 ±4.5	0.646 ±0.002	0.475 ±0.004	0.664 ±0.013
14MAY19D^3^	0	30	-66.1 ±0.6	32.8 ±1.1	-75.8 ±0.1	385.9 ±7.8	-186.8 ±5.5	0.672 ±0.015	0.520 ±0.017	0.688 ±0.022
03MAY19B^4^	0	21	-63.9 ±1.2	30.0 ±1.1	-74.3 ±1.2	325.4 ±8.5	-183.7 ±4.9	0.679 ±0.002	0.522 ±0.004	0.664 ±0.015
Mean* (paired)	0		-65.3 ±2.7	30.5 ±1.7	-76.7 ±3.1	361.4 ±27.5	-191.1 ±9.5	0.658 ±0.21	0.494 ±0.032	0.670 ±0.012
31MAY19D	0	15	-61.4 ±0.4	33.9 ±0.3	-72.8 ±0.3	420.4 ±4.1	-172.1 ±1.4	0.655 ±0.001	0.510 ±0.002	0.715 ±0.022
08MAY19B	0	20	-61.8 ±0.3	37.2 ±0.2	-71.8 ±0.4	409.9 ±11.5	-215.8 ±3.3	0.602 ±.002	0.447 ±0.003	0.620 ±0.011
Mean (all)**	0		-64.1 ±2.8	32.2 ±3.1	-75.2 ±3.3	379.3 ±35.2	-192.1 ±15.7	0.648 ±0.028	0.489 ±0.033	0.669 ±0.012
Relative change			0.964	1.003	0.984	1.108	1.147	0.889	0.864	0.887
31MAY19A^1^	300	18	-63.8 ±0.5	30.9 ±0.4	-75.5 ±0.4	394.2 ±2.2	-207.5 ±1.2	0.608 ±0.002	0.440 ±0.002	0.641 ±0.008
14MAY19A^2^	200	27	-63.0 ±0.7	34.0 ±0.5	-75.1 ±0.9	392.8 ±6.6	-221.4 ±4.7	0.588 ±0.002	0.430 ±0.005	0.598 ±0.012
14MAY19C^3^	200	15	-67.6 ±0.5	35.2 ±0.5	-76.3 ±0.5	486.8 ±7.6	-247.6 ±6.7	0.547 ±0.002	0.413 ±0.004	0.554 ±0.012
03MAY19A^4^	140	20	-62.2 ±0.5	22.3 ±0.5	-75.1 ±0.6	327.4 ±6.1	.5 ±4.7	0.595 ±0.003	0.427 ±0.005	0.585 ±0.017
Mean (X-ray)	210		-64.1 ±2.4	30.6 ±5.8	-75.5 ±0.6	400.3 ±65.6	-219.2 ±20.9	0.585 ±0.027	0.427 ±0.011	0.594 ±0.036
P-value (paired)			0.541	0.971	0.503	0.315	0.050^†^	0.005^†^	0.007^†^	0.007^†^
P-value (all)			0.969	0.587	0.859	0.524	0.046^†^	0.007^†^	0.007^†^	0.009^†^
Test/paired control			0.98 ±0.05	1.00 ±0.17	0.99 ±0.04	1.10 ±0.11	1.15 ±0.12	0.89 ±0.06	0.87 ±.08	0.89 ±0.07

**Table 4:** Effects of irradiation on the action potential in 3º giant axons with pre-synaptic simulation (anterograde propagation) Ganglion code with superscript (^1^) indicates the only experiment in which both the irradiated and (contralateral) non-irradiated stellate ganglia were tested in the same squid. All values are mean ±SD; n indicates the number of action potentials analyzed. No parameters were significantly different (p<0.05) in irradiated axons vs. control based on a two-tailed T-test SD: standard deviation

Ganglion	Dose (Gy)	n	RP (mV)	Vpos (mV)	Vneg (mV)	dV/dt Rise (V/s)	dV/dt Fall (V/s)	50% Δt (ms)	90-10% Δt (ms)	Max-Min Δt (ms)
31MAY19B^1^	0	18	-64.2 ±0.4	28.7 ±0.3	-75.0 ±0.4	367.3 ±21.6	-196.7 ±1.2	0.611 ±0.003	0.454 ±0.006	0.619 ±0.009
08MAY19B	0	19	-60.8 ±0.1	35.0 ±0.2	-68.0 ±0.4	356.1 ±2.9	-203.4 ±1.9	0.600 ±0.003	0.461 ±0.010	0.589 ±0.010
04MAY19B	0	26	-67.4 ±0.3	32.2 ±1.1	-73.2 ±0.4	355.2 ±7.4	-200.6 ±3.8	0.635 ±0.005	0.497 ±0.005	0.625 ±0.012
26APR19A	0	23	-62.4 ±0.1	36.9 ±0.5	-68.4 ±0.6	368.6 ±4.8	-193.6 ±1.9	0.643 ±0.003	0.495 ±0.004	0.639 ±0.009
Mean ±SD			-63.7 ±2.8	33.2 ±3.6	-71.2 ±3.5	361.8 ±7.2	-198.6 ±4.3	0.622 ±0.20	0.477 ±0.022	0.618 ±0.021
Relative change			1.019	0.922	1.056	1.036	1.068	0.898	0.933	0.934
31MAY19A^1^	300	21	-64.9 ±0.4	32.5 ±0.4	-74.4 ±0.4	390.8 ±2.1	-198.8 ±1.4	0.614 ±0.003	0.466 ±0.003	0.625 ±0.011
14MAY19A	200	21	-63.3 ±0.6	31.8 ±0.8	-74.7 ±0.7	332.8 ±8.4	-202.5 ±4.9	0.616 ±0.010	0.461 ±0.012	0.596 ±0.022
14MAY19C	200	15	-67.6 ±0.6	35.1 ±0.5	-75.8 ±0.4	447.7 ±9.0	-245.8 ±6.7	0.544 ±0.003	0.415 ±0.005	0.543 ±0.015
03MAY19A	140	30	-64.1 ±1.0	23.0 ±1.4	-75.9 ±0.6	328.9 ±11.5	-201.2 ±6.1	0.578 ±0.005	0.438 ±0.010	0.545 ±0.012
Mean ±SD			-64.9 ±1.9	30.6 ±5.2	-75.2 ±0.8	375.0 ±56.1	-212.1 ±22.5	0.588 ±0.034	0.445 ±0.023	0.577 ±0.040
P-value			0.496	0.441	0.064	0.657	0.283	0.134	0.094	0.121

Analysis of data for experiments with 2º stimulation (Table 4) shows no significant effect of irradiation on any parameter at p<0.05, although a 7% reduction in 90-100% time was significant at p<0.10, and the other parameters associated with action potential fall showed an effect that was in the same direction as that described above for 3º axon. In the case of 2º stimulation, Vneg also increased slightly (p<0.10).

**Figure 4:**
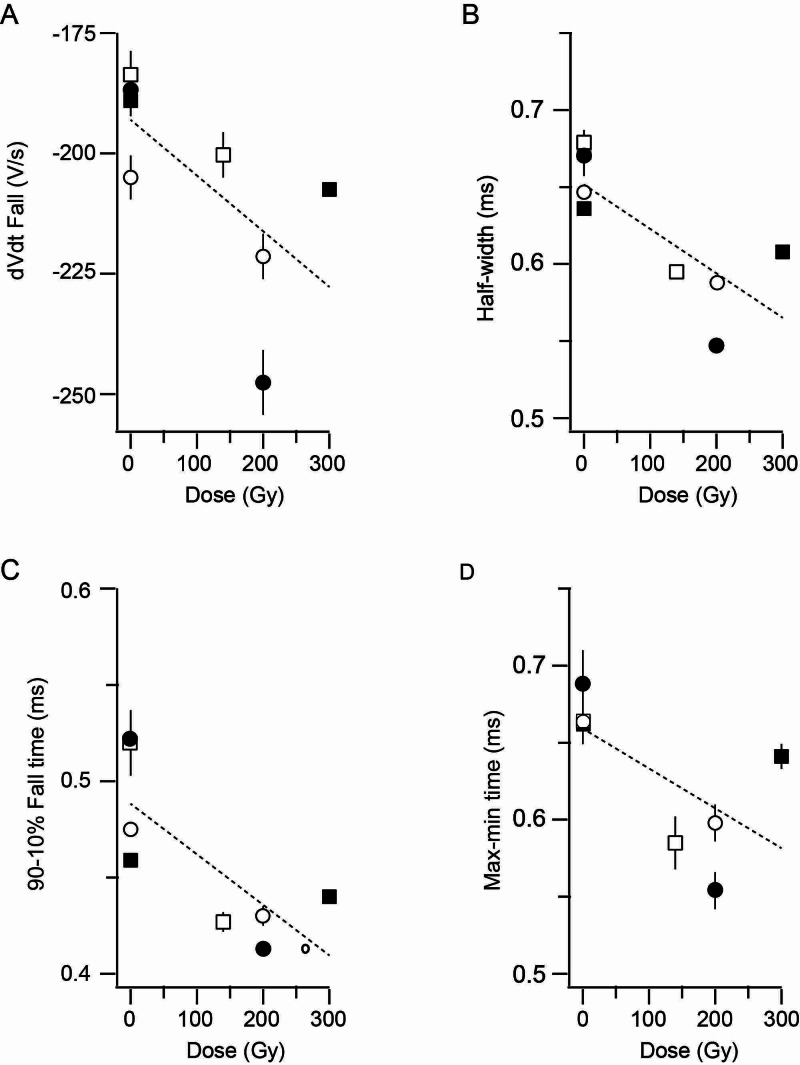
Dose-dependent effects of radiation on action potential parameters measured with stimulation of the 3⁰ giant axon Data are from the four squid in Table 3 in which both control and irradiated ganglia were successfully studied (means ±SD). Dotted lines are linear regressions. Filled squares = 31MAY1A vs. B. Open circles: 14MAY19A vs. B. Filled circles = 14MAY19C vs D. Open squares = 03MAY19A vs B A. Maximum rate of action potential fall. r^2^=0.432; p=0.077 B. Half-width of the action potential. r^2^=0.593; p=0.025 C. Time to fall from 90% to 10% of peak action potential amplitude. r^2^=0.571; p=0.030 D. Time between maximum rate of the rise and fall of the action potential. r^2^=0.436; p=0.075

Data summarized in Table 3 for stimulation of the 3º giant axon with control and irradiated ganglia successfully studied in the same squid are analyzed in terms of radiation dosage in Figure 4. Data for half-width (Figure 4B) and 90-10% rt (Figure 4C) show a significant dependence on radiation dose (p<0.05). Data for the maximum rate of fall (Figure 4A) and max-min time (Figure 4D) are marginally significant at p<0.10.

Action potential simulations

**Figure 5:**
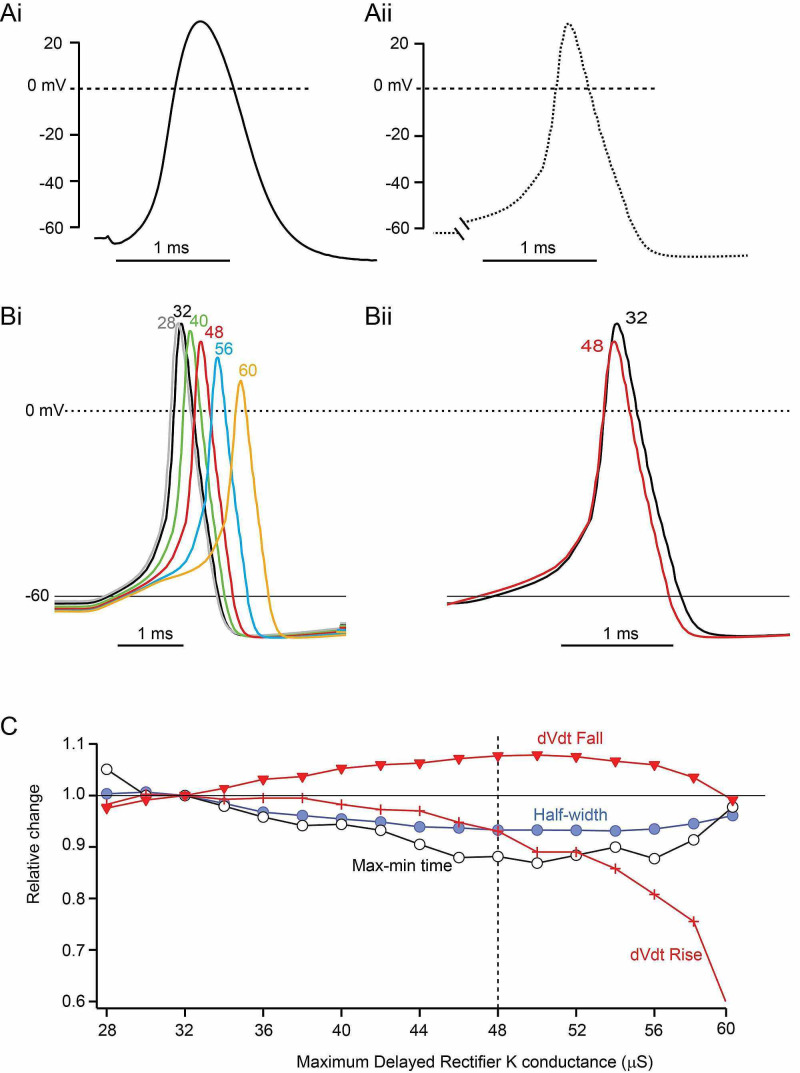
Action potential simulations based on a Hodgkin-Huxley model and the effects of increasing the maximum delayed-rectifier K conductance A. (i) Experimentally recorded action potential (3º stimulation). (ii) Action potential calculated using parameters that best matched the action potential amplitude (Vpos), the maximum rate of the rise (dV/dt Rise), and maximum rate of the fall (dV/dt Fall) B. (i) Calculated action potentials for changing the maximum level of K conductance (G_K_). Values of G_K_ are in µS. (ii) Superimposed action potentials for G_K_=32 µS and 48 µS, positioned so the action potential rising phases match C. Summary of calculated effects of parameters associated with action potentials due to varying maximum G_K_ from 28 µS to 60 µS

Action potentials were simulated using a version of the Hodgkin-Huxley equations [14] in an effort to identify a potential mechanism for the shortening of the action potential that was observed with irradiation. We adjusted the model (as described in Methods) to compute an action potential at 14 ⁰C (Figure 5Aii) that reasonably matched our recorded action potentials in regard to peak amplitude (Vpos), the maximum rate of the rise, and maximum rate of fall (Figure 5Ai). Although these parameters were in good agreement, the duration of the calculated action potential was substantially shorter, and we were not able to generate a good match for this feature. Parameters for the Hodgkin-Huxley model and their temperature dependence were derived from a different species of squid than that used in this study, and it is likely that temperature dependence of gating parameters may not be identical in the two species.

Varying the maximum amount of “delayed rectifier” potassium conductance (G_K_) produced the calculated results in Figure 5Bi. Decreasing G_K_ below a “normal” value of 32 µS led to repetitive firing for G_K_ <28 µS (not illustrated). Increasing G_K_ above 32 µS most obviously led to a progressive decline in Vpos, and the action potential failed for G_K_ >60 µS (not illustrated). In addition to the decrease in Vpos, increasing G_K_ from 30 µS to ~50 µS led to a shortening of action potential duration. This can be observed by comparing action potentials calculated for G_K_=32 µS and 48 µS with the rising phases superimposed (Figure 5Bii). This effect is qualitatively like that associated with experimental irradiation (Figure 3C).

Increasing G_K_ above 32 µS also affects other parameters associated with the falling phase of the action potential (Figure 5C). The maximum rate of fall (dV/dt Fall) increases by a maximum of almost 10% at G_K_=50 µS and then falls off. Half-width and max-min time both decrease over this same G_K_ range and then rise again. The maximum rate of the rise (dV/dt Rise) is little affected over the G_K_ range of 32 µS to 44 µS but then begins to decrease more rapidly, especially for values >50 µS. Maximum effects mimicking those observed experimentally on dV/dt Fall, half-width, and max-min time with a minimal effect of dV/dt Rise would thus be associated with an increase in maximum G_K_ from 32 µS to 48 µS (dotted line in Figure 5C).

Several other avenues of adjusting the model to mimic the effects of irradiation on the action potential were explored but with no success. These included manipulating kinetic features of G_K_ to promote more rapid activation at depolarizing voltages and of sodium conductance (G_Na_) to lead to slower activation or more rapid inactivation. Such efforts resulted in much larger effects on action potential amplitude (Vpos) and rate of the rise than on the parameters associated with the falling phase.

## Discussion

Effects of irradiation on neuronal excitability

All previous studies of acute and chronic irradiation over a wide dosage range have demonstrated generally inhibitory effects on spiking and excitatory synaptic activity in the rodent hippocampus [2-8], but analyses of changes in cellular-level parameters based on individual neurons have been somewhat limited. Recent patch-clamp work with low doses of irradiation has consistently found an increase in the resting potential of 3-6 mV [6-8] and usually a decrease in input resistance [6,8]. Both changes would be consistent with a decrease in the spiking activity, but none of the cited studies detected changes in the action potential threshold or other parameters associated with the amplitude or time course of the action potential.

Results described in this paper on squid giant axon indicate a significant (p<0.05) effect of ionizing radiation on several parameters associated with the falling phase of the action potential, specifically the maximum rate of repolarization (dV/dt Fall), half-width (50% Δt), the time to fall from 90-10% peak amplitude (90-10% Δt), and the time between the points of maximal rates of the rise and all (max-min time). Effects on resting potential, maximum rate of the rise (dV/dt Rise), overshoot (Vpos), or undershoot (Vneg) were not observed. With the methods we used, it was not possible to measure the action potential threshold, but there was no obvious change in the strength of a stimulus needed to generate an action potential in either the 2º or 3º giant axons.

Unknown biophysical or structural differences between the squid stellate ganglion and rodent hippocampus may account for the apparently differential effect of irradiation on action potentials in the two systems. However, the effects observed in squid are small, generally constituting a change of ~10% in the affected parameters, and it is possible that the inherent variability in the hippocampus, either cell-to-cell or trial-to-trial, may complicate the detection of an effect this small. Based on the published values of mean and SD for action potential half-width in hippocampal neurons, the SD:mean ratio is between ~0.1 (Table 2 in [6]) and ~0.3 (Table 2 in [7]), whereas the corresponding value from our data is ~0.04 (Table 3, paired measurements). Thus, a 10% change in half-time might not have been detectable in the hippocampus studies. Even with our smaller number of cells studied, the low variability in the case of squid confers a significant advantage.

Studies on rodent neurons reported a significant effect of radiation on the resting potential, a hyperpolarization of 3-5 mV [6-8], but we did not observe this. This comparison is somewhat difficult to consider quantitatively, because the resting potential is a complex balance that depends on the identity of multiple specific channels (and pumps) that are generally not well identified in the squid giant axon, and mammalian neurons are probably more complicated. We also did not see a change in the negative undershoot of the action potential (Vneg), a feature that is largely determined by the selectivity of the voltage-gated “delayed rectifier” potassium channels. Apparently, the highly selective nature of these channels for potassium over sodium is not altered by radiation, because even a small decrease in the relative permeability of potassium relative to sodium would be expected to change the reversal potential for the potassium channels, leasing to a more positive Vneg.

Effects of irradiation on excitatory synaptic transmission

Excitatory synaptic activity, as revealed by field measurements and recordings of spontaneous and miniature excitatory post-synaptic currents, has been consistently reported to be decreased by radiation in hippocampus studies [2-8]. Although the squid giant synapse is not a favorable preparation for the recording of small postsynaptic currents, and there is no spontaneous activity, it was clear that transmission at the giant synapse was robust in irradiated ganglia, and the refractory period of stimulation of the presynaptic 2º axon was not significantly changed (Table 2). Because our assay was recording in the postsynaptic 3º axon, and this indicates that the synapse was functioning normally, despite receiving a high dose of irradiation.

The only exception to this generalization was the single anomalous preparation in which the irradiated ganglion showed a refractory period of more than 45 seconds due to the slow recovery of the excitatory postsynaptic potential to reach the action-potential threshold in the 3º giant axon. This suggests a long-lasting depression of glutamatergic excitatory transmission at the giant synapse. The recovery time course was reliable, and many measurements were made. We never found any result like this in any other ganglia, either irradiated or control, and cannot speculate on what sort of mechanism might lead to such an effect. Nonetheless, if it is truly due to irradiation, it would be a significant alteration with great functional consequences in vivo, because the giant axon pathway often fires a brief burst of two to three action potentials ~20 ms apart during an escape response, and jet pressure (and escape acceleration) depends on the number of spikes [13,15].

Whole-body radiation of mice at a very low dose (0.5 Gy) has been reported to lead to a significant decrease in the functional synaptic connectivity between CA1 neurons and regular spiking neurons in the hippocampus, and possibly complete abolition of connectivity in the case of late spiking neurons [7]. Whether this remarkable effect is related to the abnormal synaptic transmission in our anomalous ganglion is unknown. In the case of our results, the first trial yielded an apparently normal synaptic transmission - it was only for the second stimulus that the slow recovery was observed.

Potential mechanisms for the effect of radiation on action potentials

Simulations of action potentials using a Hodgkin-Huxley model [14] pointed to an increase in the conductance of the “delayed-rectifier” potassium channel population (G_K_) as a potential mechanism for the effects of radiation on the falling phase of the action potential in the squid giant axon. Such an effect could be the result of mechanisms that act to either increase the number of functional channels in the axonal membrane or alter functional properties of existing K channels in a way that augments G_K_ at a given voltage.

Because we assayed the effects of irradiation within 24 hours, such an increase in G_K_ could be with biosynthesis or trafficking of nascent K channels. Based on estimates of the appearance of voltage-gated sodium [16] and calcium [17] in cultured giant fiber lobe neurons of the squid (the ones that form the syncytial 3⁰ giant axons), an increase in K channel density of 50%, corresponding to an increase of G_K_ from 32 to 48 µS, would appear to be easily possible within 24 hours.

Another possibility could be a modification of existing K channels, particularly by phosphorylation, a process that increases G_K_ in squid giant axons, primarily by shifting the voltage-dependence of steady-state inactivation. Voltage-clamp experiments with internally dialyzed giant axons of Loligo (Doryteuthis) pealei showed that phosphorylation increased maximal G_K_ by 20% at a holding potential of -60 mV, but the effect was highly voltage-dependent, reaching a peak of >100% at -35 mV and falling to 0% at -80 mV [18]. Our modeling results suggest a post-irradiation increase of ~50% in G_K_ at a resting potential of -64 mV (Table 1), but it is difficult to quantitatively assess this discrepancy because of differences in experimental conditions for internally dialyzed axons in voltage-clamp experiments and intact axons in our case. Nonetheless, the effects of irradiation qualitatively resemble those of phosphorylation.

Effects of phosphorylation of K channels are complex and depend on the exact subtype of K channel involved. The relevant “delayed rectifier” K channel in the squid giant axon is almost certainly a member of the Kv1 (Shaker) subfamily, but the exact subunit composition of the native channels is unknown [10]. A substantial literature on the effects of phosphorylation of Kv1 channels exists, primarily for Kv1.1 and Kv1.2, both of which are widely expressed in the nervous system [19,20]. Depending on the kinase involved, phosphorylation can either enhance or inhibit Kv1.2 channel activity, through acute modulation of functional properties, often through interaction with G-protein pathways or cytoskeletal proteins [21-24]. Enhanced biosynthesis and trafficking of channel protein to the cell membrane due to chronic phosphorylation has also been reported for Kv1.1 channels [25].

Effects of ionizing radiation on K channels

Effects of ionizing radiation on several types of K channels, though not on any of the Kv1 subtypes, have been reviewed in conjunction with oncological processes and treatments associated with human cancers [26]. In general, radiation is associated with an increase in K channel activity, and our hypothesized increase in G_K_ for a Kv1-type channel following irradiation of squid giant axons would fit with this generalization. Taken together, these observations suggest that the effects of radiation on K channels might be more widespread than appreciated.

Although K channels may play important roles in cancers and can be affected by radiation, recent reviews on the effects of ionizing radiation on the brain do not appear to even mention K channels, or any other channel types in cell membranes [27-29], although effects on the voltage-dependent anion channel (VDAC1) in the outer mitochondrial membrane have been noted [30].

## Conclusions

Effects of ionizing radiation on the rodent hippocampus have consistently shown reduced spike activity and excitatory synaptic activity, sometimes accompanied by hyperpolarization of neuronal cell bodies and a decrease in input resistance. Both features are consistent with reduced overall activity. However, no previous studies have detected specific changes in the action potentials in the cell bodies of hippocampal neurons. Radiation doses and the time to electrophysiological testing have varied widely in these studies, making quantitative comparisons difficult.

The present study investigated the effects of high doses of radiation (140-300 Gy) doses delivered focally to the squid stellate ganglion, a target that includes the giant synapse and segments from the input 2⁰ axon and output 3⁰ giant axon. Synaptic transmission was not greatly altered, except that in one irradiated ganglion a greatly increased refractory period was observed for presynaptic stimulation. A small but significant shortening of the action potential in the giant axon was detected, but changes in the resting potential or in the rising phase of the action potential were not observed. Although these changes did not prevent action potential generation or synaptic transmission, a reduced spike duration in the presynaptic axon could in principle lead to reduced glutamatergic excitation at the giant synapse.
